# Bromodomain Protein Inhibition Protects β-Cells from Cytokine-Induced Death and Dysfunction via Antagonism of NF-κB Pathway

**DOI:** 10.3390/cells13131108

**Published:** 2024-06-26

**Authors:** Vinny Negi, Jeongkyung Lee, Varun Mandi, Joseph Danvers, Ruya Liu, Eliana M. Perez-Garcia, Feng Li, Rajaganapati Jagannathan, Ping Yang, Domenic Filingeri, Amit Kumar, Ke Ma, Mousumi Moulik, Vijay K. Yechoor

**Affiliations:** 1 Diabetes and Beta Cell Biology Center, Division of Endocrinology and Metabolism, University of Pittsburgh, Pittsburgh, PA 15213, USA; negiv@pitt.edu (V.N.); jkl43@pitt.edu (J.L.); mandi.varun@medstudent.pitt.edu (V.M.); ruya.liu@som.umaryland.edu (R.L.); emperezgarcia@southalabama.edu (E.M.P.-G.); fel43@pitt.edu (F.L.); domfil@pitt.edu (D.F.); amk550@pitt.edu (A.K.); 2 Division of Cardiology, Department of Pediatrics, Children’s Hospital of Pittsburgh, University of Pittsburgh, Pittsburgh, PA 15224, USA; jagan@pitt.edu (R.J.); moulikm@pitt.edu (M.M.); 3 Department of Diabetes Complications and Metabolism, Diabetes and Metabolism Research Institute, City of Hope National Medical Center, Duarte, CA 91010, USA; kema@coh.org

**Keywords:** Brd4, bromodomain, I-BET, islet, β-cells, insulin, NF-kB, inflammation, STZ, diabetes, apoptosis, cytokines

## Abstract

Cytokine-induced β-cell apoptosis is a major pathogenic mechanism in type 1 diabetes (T1D). Despite significant advances in understanding its underlying mechanisms, few drugs have been translated to protect β-cells in T1D. Epigenetic modulators such as bromodomain-containing BET (bromo- and extra-terminal) proteins are important regulators of immune responses. Pre-clinical studies have demonstrated a protective effect of BET inhibitors in an NOD (non-obese diabetes) mouse model of T1D. However, the effect of BET protein inhibition on β-cell function in response to cytokines is unknown. Here, we demonstrate that I-BET, a BET protein inhibitor, protected β-cells from cytokine-induced dysfunction and death. In vivo administration of I-BET to mice exposed to low-dose STZ (streptozotocin), a model of T1D, significantly reduced β-cell apoptosis, suggesting a cytoprotective function. Mechanistically, I-BET treatment inhibited cytokine-induced NF-kB signaling and enhanced FOXO1-mediated anti-oxidant response in β-cells. RNA-Seq analysis revealed that I-BET treatment also suppressed pathways involved in apoptosis while maintaining the expression of genes critical for β-cell function, such as Pdx1 and Ins1. Taken together, this study demonstrates that I-BET is effective in protecting β-cells from cytokine-induced dysfunction and apoptosis, and targeting BET proteins could have potential therapeutic value in preserving β-cell functional mass in T1D.

## 1. Introduction

Type 1 diabetes (T1D) is an autoimmune disorder characterized by the destruction of insulin-producing β-cells [1,2,3]. At the molecular level, inflammatory cytokine leads to β-cell dysfunction with impaired glucose-stimulated insulin secretion (GSIS), reduced proliferation, and increased apoptosis. While a pancreas or islet transplant leads to a ‘cure’ and insulin-free periods, these approaches remain challenging due to limited donor availability and the need for chronic immunosuppression [4,5]. GLP-1 receptor agonists have been shown to protect β-cells from cytokine-induced apoptosis and improve β-cells’ proliferation and function [6,7,8]. Although it is approved to be used only in patients with T2D, and not T1D, some studies show benefits with potential risks with GLP-1R agonists in T1D [9,10,11,12]. Hence, insulin replacement remains the standard of care to date, although long-term injections’ difficulty to maintain good glycemic control makes insulin therapy suboptimal. Replacing β-cells with those derived from patient-specific iPS (induced pluripotent stem) cells remains in experimental or early clinical trial stages and has yet to effectively overcome the hurdle of cytokine-induced apoptosis in an autoimmune setting [13,14,15]. Thus, there is an imperative need to develop therapeutics for T1D that protect β-cells from cytokine-induced cell death with the preservation of β-cell mass. 

The bromodomain-containing BET (bromodomain and extra-terminal domain-containing) proteins, including Brd2, Brd3, Brd4, and Brdt, are epigenetic regulators of gene transcription known to be involved in various physiological processes, including inflammatory response, proliferation, apoptosis, and differentiation [16,17]. These proteins bind to the acetylated lysine of histones in the genome, acting as a ‘reader’ and recruiting histone remodeling proteins, such as HATs (histone acetyltransferase), HDACs (histone deacetylase), and transcription elongation factors [17,18]. Recently, with the advent of a new generation of higher-affinity chemical inhibitors such as I-BET, there has been a surge in testing their therapeutic utility for many oncological (prostate, breast, hematopoietic glioblastoma) and cardiovascular diseases despite the limited knowledge of underlying molecular mechanisms [19,20]. In diabetic NOD mice, I-BET, which primarily targets Brd2, 3, and 4 [16,21], was reported to promote an anti-inflammatory phenotype of infiltrating pancreatic macrophages and enhance β-cell proliferation [22] with reduced SASP (senescence-associated secretory phenotype) response [23]. However, the cell-autonomous function of bromodomain protein inhibition in β-cells has not been addressed. Notably, BET inhibition by JQ1 in INS-1 β-cells increased insulin content and secretion, though this was assessed under basal conditions in the absence of the autoimmune milieu to mimic disease condition [24]. Thus, whether the inhibition of BET proteins in β-cells could confer protection against cytokine-induced injury in T1D remains unclear. To know its applicability, it is crucial to determine the effect of I-BET on cytokine-induced dysfunction in a time-dependent context. To understand its potential therapeutic potential in T1D, determining BET inhibition in protecting β-cell function and identifying the molecular mechanisms involved in a model mimicking T1D may provide a mechanistic basis for potential therapeutic applications. 

In this study, we tested I-BET762 ((4S)-6-(4-Chlorophenyl)-N-ethyl-8-methoxy-1-methyl-4H-[1,2,4]triazolo[4,3-a][1,4]benzodiazepine-4-acetamide), referred to as I-BET, as a representative BET protein inhibitor currently in clinical trials [19,25] for its potential protection of β-cells at early and later time points. Employing both in vitro and in vivo approaches using a low-dose STZ model to mimic T1D, we specifically determined whether I-BET is beneficial against cytokine-induced β-cell apoptosis and dysfunction. Mechanistically, we use global transcriptomic analysis to uncover the regulation of I-BET on augmenting factors critical to maintaining β-cell function and antagonizing the cytokine induction of the NF-kB-mediated apoptotic pathway in pancreatic β-cells [26].

## 2. Research Design and Methods

Cell culture—rat insulinoma; INS-1 (832/13 cells were a gift from Dr. Christopher Newgard) cells were cultured in RPMI-1640 medium containing 11 mM glucose, 10% fetal bovine serum, 10 mM Hepes, 2 mM L-glutamine, 1 mM sodium pyruvate, and 0.05 mM 2-mercaptoethanol. The cells were pre-treated with I-BET762 (500 nM, SelleckChem, Houstan, TX, USA) or vehicle control (VC) for 48 h followed by the addition of a cytokine cocktail (CC) of IL-1β (10 ng/mL), IFN-γ (100 ng/mL), and TNF-α (25 ng/mL) (Peprotech, Cranbury, NJ, USA) for another 8 h or 24 h as indicated in the presence of I-BET or VC.

Annexin-PI apoptosis assay—INS-1 cells, after treatment as indicated, were trypsinized and stained with Annexin-V (labeled APC) and PI for 15 min at room temperature in the dark and assessed on flow analyzer BD LSR II using BD FACSDiva^TM^ software (https://www.bdbiosciences.com/en-us/products/software/instrument-software/bd-facsdiva-software, (accessed on 10 June 2024)) and analyzed on FlowJo (Version 10).

RNA isolation and real-time PCR—RNA was isolated using Direct-zol RNA miniprep kits from Zymo Research according to the manufacturer’s instructions and quantified using Nanodrop. The cDNA was synthesized using an amfiRivert kit from GenDEPOT, Baker, TX, USA according to the manufacturer’s instructions using oligo(dT) primers. The qRT-PCR was performed using an amfiSure qGreen master mix from GenDEPOT according to the manufacturer’s instructions on Quant Studio 3 from ThermoFisher Scientific, Waltham, MA, USA. All the primers used are listed in Supplementary Table S1. In preliminary experiments, qPCR was performed with many housekeeping genes, including ribosomal gene r36b4, Gapdh, beta-Actin, and Top I. Under the conditions of the experiments with the INS-1 cells, r36b4 was the most stable among the genes tested in all the groups. The primers for qPCR were optimized using a melting curve. The relative gene expression was calculated using the ddCt method using ribosomal gene r36b4 as a normalization control. The fold change was calculated over VC.

Glucose-stimulated insulin secretion (GSIS) and insulin content determination—GSIS was performed as previously reported [27]. Briefly, INS-1 cells were treated with I-BET (IB) and CC as indicated and incubated in KRB (Krebs Ringer Bicarbonate) buffer with no glucose for 30 min at 37 °C with CC and I-BET/VC, followed by serial incubations for 30 min in 2.8 mM, 11.1 mM, and 25 mM glucose in KRB. The supernatant was collected for an insulin secretion assay. At the end of the experiment, to determine cellular insulin content, the cells were lysed with acid–ethanol lysis buffer overnight at 4 °C, and the supernatant was collected after centrifugation at 16,000 rcf for 30 min. Insulin secretion and content were determined with the insulin ELISA kit (Crystalchem, Elk Grove Village, IL, USA) according to the manufacturer’s instructions and normalized to the DNA content. This was repeated three times with 4-5 biological replicates each time.

RNA-Seq—the integrity and purity of isolated RNA were analyzed using an RNA Nano 6000 Assay Kit of the Bioanalyzer 2100 system (Agilent Technologies, Santa Clara, CA, USA). A total of 1 µg RNA per sample was used as input material for sample preparations. Sequencing libraries were generated using NEBNext^®^Ultra™RNA Library Prep Kit for Illumina^®^ (NEB, Ipswich, MA, USA) sequenced on an Illumina platform (Novaseq 600) according to the manufacturer’s instructions, and at least 60 million reads of 125 bp paired-end reads were generated per sample. The raw data obtained were filtered, cleaned, and aligned to the genome using HISAT2. The gene expression was calculated as FPKM, and differentially expressed genes across all the samples with p_adj_ < 0.05 were represented in a heatmap. The gene overlap between the two groups was determined using Oliveros, J.C. (2007–2015) Venny. Gene set enrichment analysis (GSEA) [28,29] was performed among CCVC (CC + VC) and CCIB (CC + I-BET). Pathway enrichment was determined by using DAVID bioinformatic resources 6.8 [30,31] and QIAGEN IPA [32].

Western blotting—Western blotting was performed as described in [33]. Briefly, lysates were prepared in ice-cold 1× lysis buffer (Thermo Fisher Scientific, Waltham, MA, USA) supplemented with 1× protease inhibitor and phosphatase inhibitor (Roche, Indianapolis, IN, USA). An equal amount of protein samples was loaded and fractionated by SDS-PAGE and then transferred to 0.2 μm pore-size nitrocellulose membranes (Millipore Sigma, St. Louis, MO, USA). Membranes were blocked in 5% non-fat milk–TBS (*w/v*) and then incubated with indicated primary antibodies—cleaved caspase-3 (CST, Danvers, MA, USA, 9664), Pdx1 (Abcam, Waltham, MA, USA, ab47308), phospho-p65, p65, p-IKKA/B, IKKB, p-IκBα, and IκBα (CST, Danvers, MA, USA, 9936)—overnight at 4 °C and later with DyLight 680 or DyLight 800—conjugated secondary antibodies. The blots were imaged using Licor Odyssey Clx and quantified by Image Studio ver 5.2 software.

Mouse studies—all animal experiments were approved by the Institutional Animal Care and Use Committee of the University of Pittsburgh. Eight-week-old C57BL/6N mice were purchased from Jackson Laboratory, Bar Harbor, ME, USA. For the induction of diabetes, 50 mg/kg of streptozotocin (STZ), freshly dissolved in Na-citrate buffer at pH 4.2, was administered via i.p. for five days. The mice were distributed into three groups: VC—non-diabetic controls (sodium citrate buffer only), STZ + VC—STZ with vehicle control for I-BET, and STZ + I-BET, with n = 9 in each group. The drug I-BET or its vehicle control (2% DMSO) was given daily orally by gavage for four weeks. Note that 2% DMSO is way below the well-tolerated dose in mice, thus safe to administer [34]. The body weight and blood glucose levels of all the mice were monitored every week for four weeks. A glucose tolerance test (GTT) was performed on days 26 and 27 in overnight-fasted mice by administering 1.5 g/kg of body weight by i.p. of D-glucose. Mice were euthanized on day 28, and the pancreases were collected. The blood glucose was measured using a glucometer and by Infinity glucose hexokinase from Thermo Fisher Scientific according to the manufacturer’s instructions.

Immunofluorescence staining—mice’s pancreatic tissue fixed in formaldehyde was embedded in paraffin and sectioned at 5 µm thickness. All the sections were stained, as previously reported [33]. Briefly, slides were warmed for 20 min at 60 °C, followed by deparaffinization using citrasolv and then rehydrating with decreasing ethanol concentrations. This was followed by permeabilization in 0.25% of Triton-x-100 in PBST for 5 min and antigen retrieval in sodium citrate buffer pH 6 in a microwave (1 min at maximum power and then 20 min at minimum power). After cooling down to room temperature, they were blocked with 1% BSA in 1× PBS and incubated with primary antibodies against cleaved caspase-3 (CST-9661, 1:100) and INS-1 (Abcam-ab6995, 1:400) overnight at 4 °C and subsequently labeled with secondary antibodies for 1 h at room temperature. All the images were taken using the Nikon (Melville, NY, USA) fluorescence microscope.

β-cell area determination—two slides per mouse, at least 50 µm apart, were selected to stain for insulin. Images of whole sections were taken by the automated EVOS FL Auto 2 imaging system. Each image’s insulin-positive area was measured and divided by the total pancreatic area using ImageJ (https://imagej.net/ij/download.html, (accessed on 10 June 2024)). The size of the islets was categorized into small, medium, and large based on their arbitrary area as computed by ImageJ—less than 10,000 (small), 10,000-30,000 (medium), and greater than 30,000 (large).

Statistical analysis—the data are presented as means ± sem; the in vitro data are representative of three independent experiments. Significance tests were determined by one-way or two-way ANOVA, followed by Sidak’s multiple comparisons test. *p* ≤ 0.05 was considered statistically significant. GraphPad Prism 8.3.1 software was used for statistical analyses.

Data and resource availability—the RNA-Seq raw and processed datasets were deposited in the GEO database (GSE160573). The differentially expressed gene list among all four groups is in Supplementary Table S2.

## 3. Results

### 3.1. I-BET Protects against Cytokine-Induced β-Cell Dysfunction and Apoptosis

**Figure 1:**
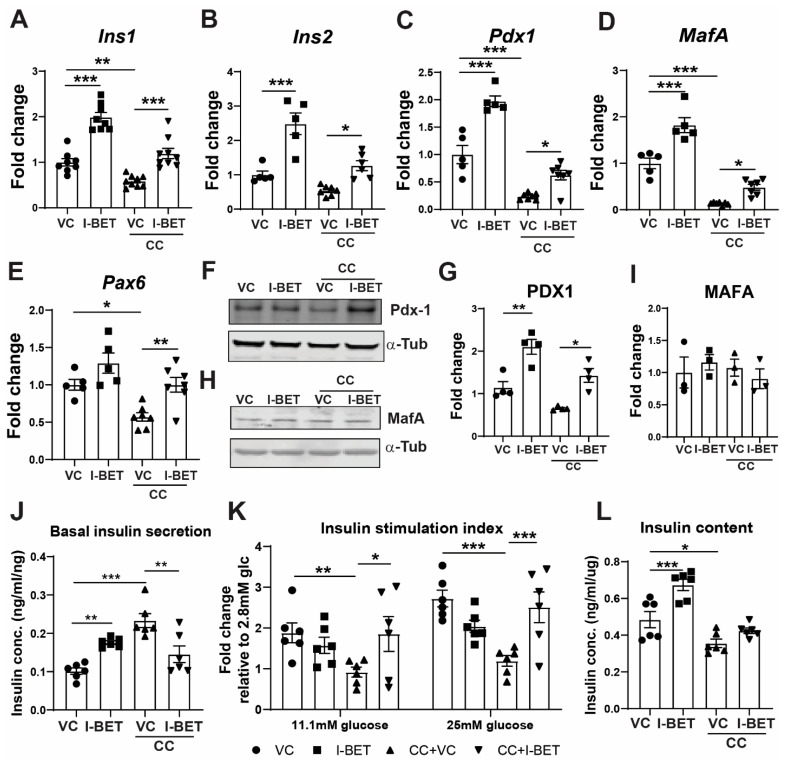
I-BET protects against cytokine-induced β-cell dysfunction. INS-1 cells were pre-treated with I-BET or VC (vehicle control) for 48 h, and then a cytokine cocktail (CC) was added for another 8 h. (**A**–**E**) Gene expressions by RT-qPCR of (**A**) *Ins1*, (**B**) *Ins2*, (**C**) *Pdx1*, (**D**) *MafA*, and (**E**) *Pax6* are shown after normalization to the housekeeping gene as a fold change over VC. (**F**–**I**) The protein level by Western blotting and the quantification by densitometry of 4 independent experiments are shown for Pdx1 (**F**,**G**) and MafA (**H**,**I**) and represented as a fold change over VC. α-Tubulin was used as a loading control. (**J**,**K**) The secreted insulin from INS-1 cells in basal 2.8 mM glucose (**J**) and after incubation in indicated glucose concentrations (**K**) is shown. Insulin secretion is represented as an insulin stimulation index (**K**) as a fold change over the respective levels from the basal 2.8 mM glucose. (**L**) Insulin content is measured in INS-1 cell lysates normalized to cellular DNA. The data are represented as mean ± sem (n = 3–7) with at least three independent experiments. Statistical significance was calculated using one-way ANOVA; *** *p* < 0.001, ** *p* < 0.01, * *p* < 0.05.

I-BET has been shown to be beneficial in the NOD model of T1D with its effect on NF-kB signaling in leucocytes [22]. We wanted to test its effectiveness on some early and late cytokine-induced β-cell dysfunction changes. Cytokine-induced β-cell dysfunction with impaired insulin secretion and cell death is the defining feature of T1D. To mimic this in vitro, INS-1 cells were exposed to a cytokine cocktail (CC) of IL-1β (10 ng/mL), IFN-γ (100 ng/mL), and TNF-α (25 ng/mL) for 8 h and 24 h to determine early and late changes, respectively. I-BET or DMSO vehicle control (VC) was administered to INS-1 cells for 48 h, followed by cytokine exposure [24,35]. Gene expression analysis revealed that I-BET significantly up-regulated genes critical for β-cell function, including *Ins1*, *Ins2*, and transcription factors critical to maintaining mature identity, *Pdx1*, *MafA*, and *Pax6*. More importantly, it prevented the repression of these genes induced by cytokine exposure (Figure 1A–E), suggesting cytoprotective effects of I-BET on β-cells against cytokine challenge. Notably, I-BET largely restored the expression levels of these genes to a comparable level to control cells without cytokine exposure. The increase in the *Pdx1* transcript was also reflected in a consistent increase in its protein level with I-BET treatment (Figure 1F,G). Interestingly, despite increased MafA mRNA, its protein level was not altered by cytokine or I-BET, suggesting the potential involvement of post-translational modifications and MafA protein stability regulation [36,37] (Figure 1H,I). In comparison, genes such as *Pax4, Nkx2.2*, and *Nkx6.1* did not respond to I-BET treatment (Figure S1A–C), suggesting the potential specificity of gene regulation by BET proteins. We then assessed if these gene regulations induced by I-BET led to functional improvement in GSIS. Under basal (2.8 mM glucose) conditions, insulin secretion was increased by both I-BET and cytokines (Figure 1J). The increase in basal insulin secretion on exposure to cytokines is indicative of a defect in regulated insulin secretion and is consistent with previous reports [38,39,40]. More importantly, and in concordance with the enhanced expression of *Pdx1* and *Ins-1*, I-BET prevented the cytokine-induced reduction of the insulin secretion index (Figure 1K). Total cellular insulin content was increased by pre-treatment with I-BET, consistent with prior reports [24], although this effect was lost in the presence of cytokines (Figure 1L). At this early 8 h time point, no significant difference in apoptosis (AnnexinV-PI positive cells) was observed in cells induced with cytokines compared to controls (Figure S1D,E).

**Figure 2:**
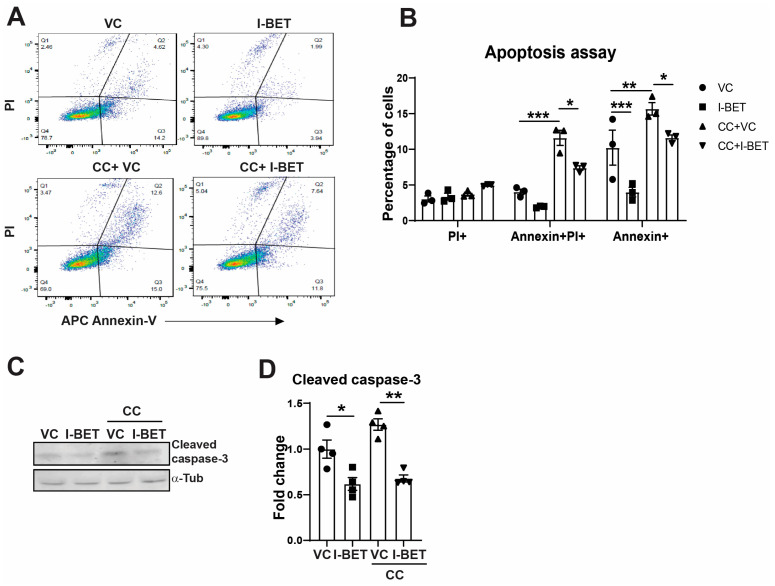
I-BET protects against cytokine-induced β-cell apoptosis in vitro. (**A**,**B**) INS-1 cells pre-treated with I-BET or VC (vehicle control) for 48 h and then exposed to a cytokine cocktail for another 24 h were evaluated by PI and AnnexinV staining by flow cytometry with appropriate controls. The representative dot plot is shown in (**A**) for the four groups, and the quantitation of early (Annexin+) and late (Annexin+ PI+) apoptotic cells from three independent experiments is shown in (**B**). (**C**,**D**) The protein level of caspase-3 is shown by Western blotting, and the quantification by densitometry of 4 independent experiments is represented as a fold change over VC. α-Tubulin was used as a loading control. The data are described as mean ± sem (n = 3–4) with at least three independent experiments. Statistical significance was calculated using two-way (**B**) or one-way ANOVA (**D**); *** *p* < 0.001, ** *p* < 0.01, * *p* < 0.05.

However, at 24 h of cytokine exposure, I-BET could not improve β-cell function as determined by gene expression and GSIS (Figure S2A–G). At the 24 h time point, cells started to die with a significant increase in AnnexinV-PI positive cells, with ~15% cells showing early apoptosis (Annexin V^+^, PI^−^), ~12.5% with late apoptosis (Annexin V^+^, PI^+^), and ~4% dead cells (PI^+^) (Figure 2A,B). Importantly, I-BET significantly reduced the percentage of early (Annexin V^+^, PI^−^) and late apoptotic cells (Annexin V^+^, PI^+^) after cytokine exposure in I-BET-treated cells as compared to controls, without a significant change in PI^+^ cells, indicating that I-BET protects against cytokine-induced β-cell apoptosis. Consistent with this, I-BET markedly decreased the cleaved caspase-3 protein levels after cytokine exposure (Figure 2C,D) in Western blotting. These data demonstrate that I-BET pre-treatment protected β-cells from the deleterious effects of cytokines at an early time point and from apoptosis at a later time point, though it was not sufficient to prevent β-cell insulin secretion from chronic exposure of cytokines.

### 3.2. I-BET’s Effect on Protecting β-Cells from Cytokine-Mediated Apoptosis Is Mediated by Antagonizing the NF-kB Signaling Pathway

**Figure 3:**
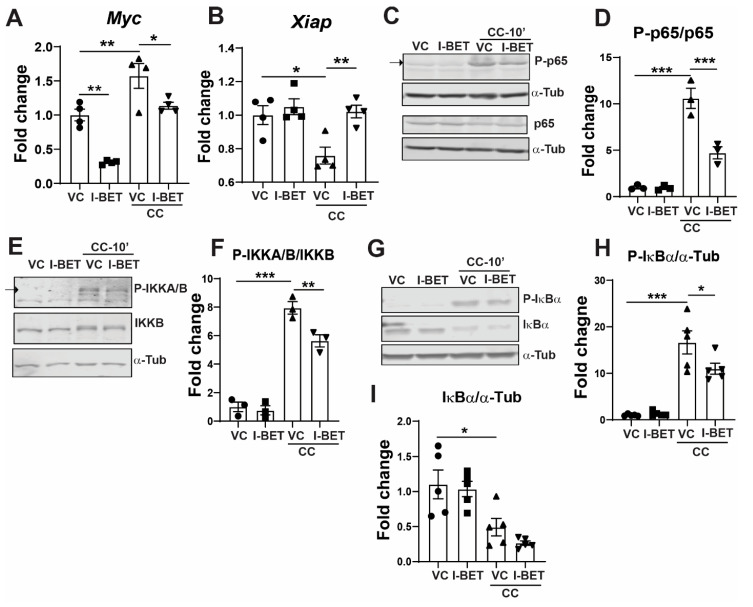
I-BET mediates its effect by antagonizing NF-kB pathway activation. (**A**,**B**) The expression of NF-kB target genes—*Myc* and *Xiap*—by RT-qPCR shown after normalization to the housekeeping gene as a fold change over VC. (**C**–I) The phospho-protein and protein level by Western blotting and the quantification by densitometry of 3-4 independent experiments are shown for p65 (**C**,**D**), IKKA/B (**E**,**F**), and IκBα (**G**–**I**). α-Tubulin was used as a loading control. Quantitation from densitometry is represented as a fold change over VC. The data are described as mean ± sem (n = 3–4), with at least three independent experiments. Statistical significance was calculated using one-way ANOVA; *** *p* < 0.001, ** *p* < 0.01, * *p* <0.05.

NF-κB is one of the major pathways responsible for inflammation-induced apoptosis of pancreatic β-cells [41,42], and inhibiting this could provide a therapeutic modality to preserve β-cells in T1D. Since Brd4 has been shown to interact with p65 [43], we wanted to determine the effect of I-BET on NF-κB pathway activation. Furthermore, since I-BET is known to affect the NF-kB signaling pathway [41,42], we tested if NF-κB signaling and targets were changed by I-BET in cytokine-stimulated INS-1 cells. We found that the expressions of its downstream targets—*Myc* [44] and *Xiap* [45], which are up- and down-regulated by NF-κB upon cytokine stimulation, respectively—were reversed by I-BET (Figure 3A,B). Furthermore, p65 (RelA), the subunit of NF-κB transcription factor, translocates to the nucleus upon cytokine-stimulated phosphorylation and binds to the promoters/enhancers to induce the expression of cytokine target genes. Hence, we tested if I-BET altered NF-κB signaling by examining the phosphorylation of p65 and found that it was markedly decreased by I-BET in CC-treated INS-1 cells (Figure 3C,D). Furthermore, upstream regulators of p65 include the activation, and the phosphorylation of IKKA/B, which leads to phosphorylation and degradation of IκBα, relieving the inhibition on p65 and allowing it to translocate into the nucleus [46]. We found that I-BET treatment also reduced the phosphorylation of IKK and IκBα in cytokine-exposed cells (Figure 3E–I), indicating an overall suppression of cytokine-induced NF-kB pathway activation. It is worth noting, however, that I-BET did not completely abolish cytokine-induced NF-kB activation, as phospho-p65 remained elevated compared to the level observed in unstimulated INS-1 cells.

### 3.3. Global Transcriptome Profile Reveals Enrichment of Pathways Related to β-Cell Function with BET Inhibition

**Figure 4:**
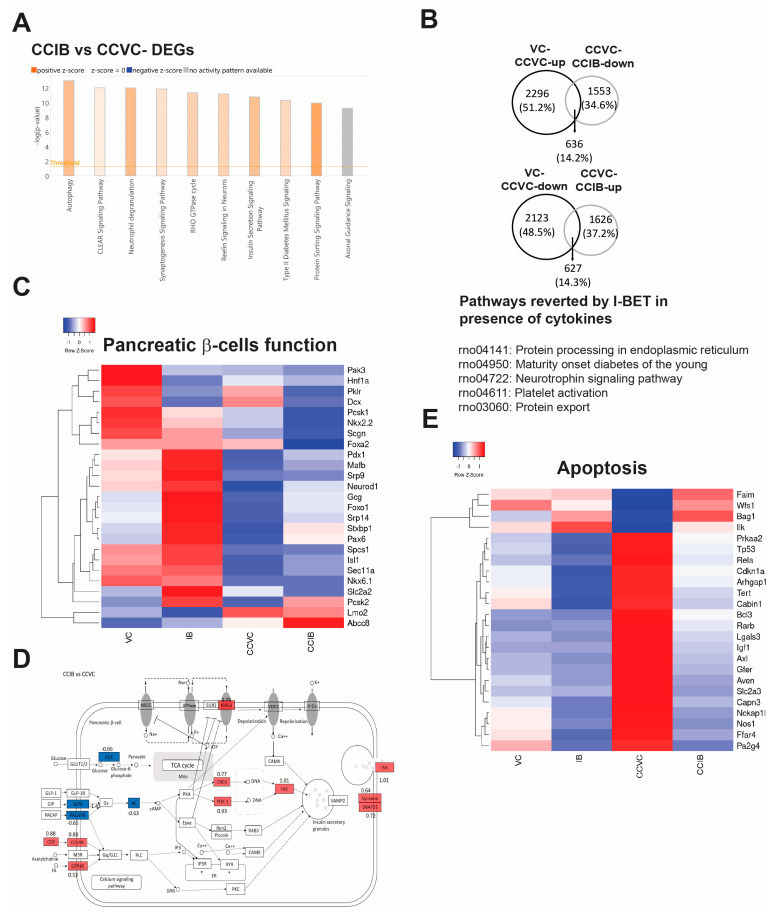
Global transcriptomic changes after I-BET rescues the cytokine-induced changes in INS-1 cells. RNA-Seq analysis from INS-1 cells treated with I-BET or vehicle control (VC) under short (8 h) exposure to the cytokine cocktail (CC). (**A**) The top 10 enriched pathways of differentially expressed genes (DEGs) between CCIB and CCVC, as determined by IPA. The colors orange and grey depict positive and zero z-scores, respectively. The shades of orange represent stronger correlation with deeper color. (**B**) The Venn diagram illustrates the genes that were reverted by I-BET in the presence of cytokines. Subsequently, we have the top 5 enriched pathways from those genes as determined by DAVID analysis. (**C**) A heatmap of pancreatic β-cell function genes that were reverted by I-BET with cytokines. Red and blue indicate up- and down-regulated Z-score-normalized gene expression. (**D**) The insulin secretion pathway was also among the differentially regulated pathways between CCIB and CCVC. Red and blue represent the up- and down-regulated genes based on their log2 fold change. (**E**) A heatmap of apoptosis-relevant genes regulated by I-BET, where red and blue indicate up- and down-regulated Z-score-normalized gene expression.

The data demonstrated that I-BET was able to protect β-cells from cytokine-mediated cell dysfunction. To investigate pathways related to β-cell function that are regulated by BET inhibition and underpin this protection in addition to the NF-kB pathway, we performed RNA-Seq analysis in cells exposed to cytokines for 8 h with and without I-BET treatment. Among the four groups—VC (vehicle control), IB (I-BET762), CCVC (cytokine cocktail + vehicle control), and CCIB (cytokine cocktail+I-BET762)—9694 genes were differentially expressed (Figure S3A). Pathway analysis revealed that genes related to autophagy, insulin secretion signaling, T2D signaling, and protein sorting were up-regulated with I-BET treatment in the basal state (Figure 4A). Further analysis revealed that 2932 genes were up-regulated and 2750 down-regulated by cytokines (CC+VC) compared to VC, while 2189 and 2253 genes were up- and down-regulated by BET inhibition in cells exposed to cytokines (CCIB vs. CCVC), with 2343 (~30%) genes differentially regulated in both comparison groups (Figure S3B).

To determine the pathways that could explain the protection accorded by I-BET to cytokine-exposed cells, we sought to identify genes and enriched pathways that were reverted towards controls by I-BET treatment in cytokine-exposed cells. Notably, 14% of both the up-and down-regulated genes were reverted by I-BET treatment (Figure 4B). Interestingly, ER processing and MODY (maturity-onset diabetes of the young)-related pathways were among the highly enriched pathways that were reversed by I-BET treatment. A detailed examination of pathways related to β-cell function, insulin secretion, and apoptosis revealed that many genes that were altered by cytokines were restored by I-BET treatment. These included genes such as *Pdx1*, *Neurod1*, *Foxo1*, *Isl1*, and *Pax6*, critical for mature β-cell function (Figure 4C), and genes involved in insulin secretion, including *Ins1, Ins2, Pdx1, Syntaxin, SNAP25, and Creb* (Figure 4D), congruent with the observed changes in gene expression by qPCR and GSIS induced by I-BET (Figure 1). Consistent with the observed protection against cytokine-induced apoptosis, many genes related to apoptosis, significantly up-regulated by cytokines, were down-regulated by I-BET treatment (Figure 4E). Taken together, the global transcriptomic analysis reveals an enrichment of pathways that enhance β-cell function and protect against apoptosis in the presence of cytokines with BET inhibition. 

**Figure 5:**
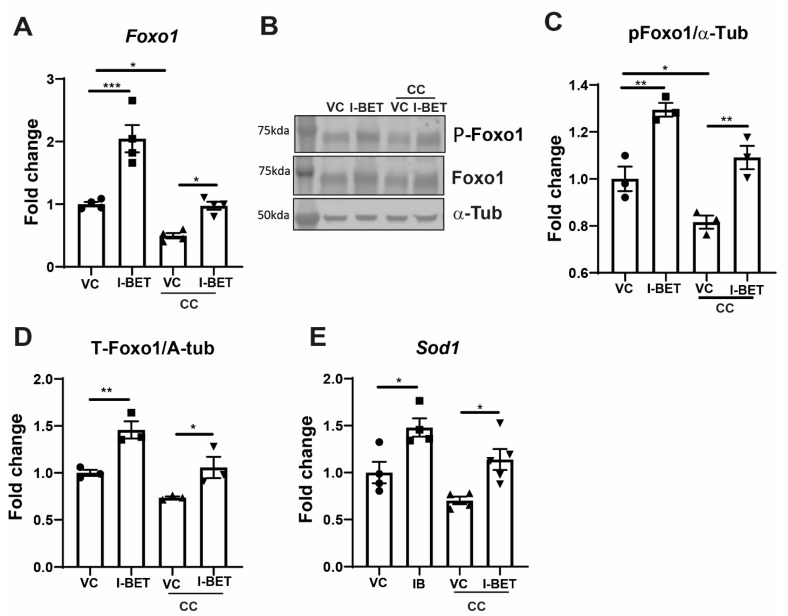
I-BET prevents cytokine-mediated suppression of Foxo1. (**A**) The expression of *Foxo1* mRNA in INS-1 cells was induced by cytokine cocktails (CCs) in 8 h as determined by qRT-PCR and normalized by loading control with respect to VC. (**B**–**D**) The protein level of phosphorylated Foxo1 and total Foxo1, along with loading control (alpha-tubulin (α-Tub)) is shown in (**B**). Its respective densitometry is represented in (**C**,**D**). (**E**) The Foxo1 target gene *Sod1*’s expression was measured in the same condition using qRT-PCR and normalized by loading control and VC. The data are represented as mean ± sem (n = 3–4), with at least three independent experiments. Statistical significance was calculated using one-way ANOVA; *** *p* < 0.001, ** *p* < 0.01, * *p* < 0.05.

Cytokines induce an oxidative stress response in pancreatic β-cells that reduces Foxo1 levels [47], and I-BET is known to have an anti-oxidative effect on other cells [48]. We wanted to know if I-BET could reduce the oxidative stress response by modulating Foxo1 and its downstream target SOD1. *Foxo1*’s gene expression was found to be lowered with cytokines, and I-BET alone increased its expression and protected it from cytokine damage (Figure 5A). Activated phosphorylated Foxo1 also decreased with cytokines, as the total Foxo1 was determined by Western blotting (Figure 5B–D), as shown by the representative blot and densitometry. Foxo1 is known for its anti-oxidant and anti-apoptotic role [49], so we determined its target superoxide dismutase (SOD1) gene, a known anti-oxidant. SOD1 followed Foxo1 expression, which decreased with cytokines and was protected by I-BET (Figure 5E).

### 3.4. I-BET Protects against Cytokine-Induced Apoptosis in the Multiple Low-Dose STZ Diabetes Mice Model

**Figure 6:**
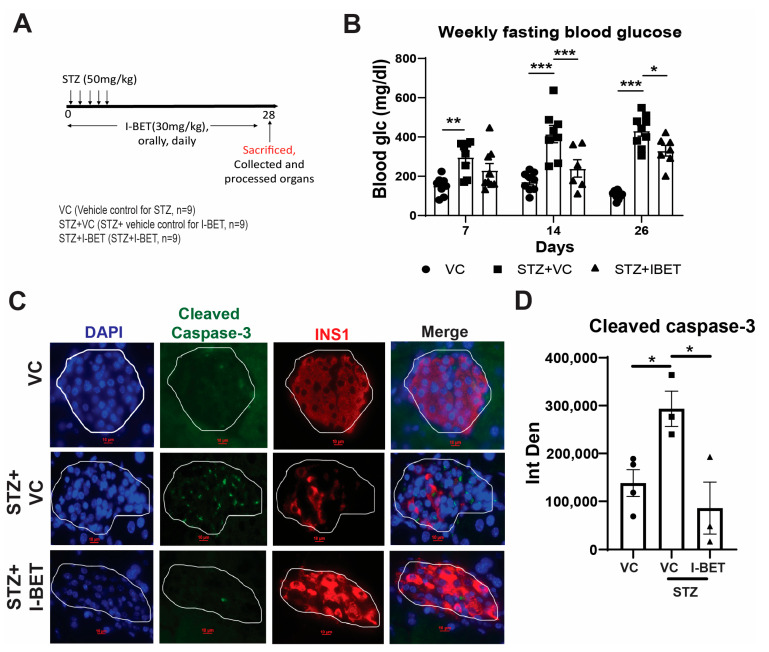
I-BET protects against cytokine-induced β-cell apoptosis in vivo. (**A**) An experimental scheme using the multiple low-dose STZ mouse model was used. (**B**) Fasting blood glucose level (n = 9 mice/group). (**C**,**D**) Representative sections (**C**) from the pancreas stained for cleaved caspase-3 (green), insulin (red), and DAPI (blue) are shown. The scale bar is 10 µm. The quantitation of cleaved caspase-3 signal from insulin-positive β-cells in each islet is shown (**D**) with n = 4 mice/group with 2-3 slides for each mouse. The data are represented as mean ± sem. Statistical significance was calculated using two-way ANOVA; *** *p* < 0.001, ** *p* < 0.01, * *p* < 0.05.

The above experiments demonstrated that I-BET inhibits the cytokine-induced apoptosis of pancreatic β-cells in vitro. We further determined if this protection applies to a well-established T1D model induced by multiple low-dose STZ injections [50]. STZ enters the pancreatic β-cells by Glut-2 transporter and causes DNA damage and subsequent apoptosis of β-cells. When administered at a higher dose of 100–200 mg/kg body weight, STZ-induced β-cell apoptosis is rapid and leads to profound diabetes. However, a multiple low-dose STZ regimen (30–50 mg/kg body weight daily for 5 days), often used to model immune-mediated T1D, induces oxidative stress, DNA damage, and an inflammatory reaction leading to a more gradual β-cell death [51,52]. Hence, we used this model of immune-mediated diabetes to study the anti-apoptotic effect of I-BET. I-BET or vehicle control (VC) was administered (30 mg/Kg by gavage once daily) for four weeks to mice that received low-dose daily STZ five times at 50 mg/kg (Figure 6A). Weekly fasting blood glucose levels were measured to assess the effect of I-BET treatment, and strikingly, we observed a significant improvement in fasting hyperglycemia in the I-BET-treated group (Figure 6B). The body weight and fasting insulin levels of I-BET-treated mice (STZ + I-BET), however, did not differ from those of the control STZ group (STZ + VC), which were reduced as compared to vehicle control (VC), as expected (Figure S4A,B). The glucose tolerance test revealed significantly lower glucose levels at 30 min following glucose injection in the STZ+I-BET compared to STZ + VC (Figure S4C). However, insulin levels did not differ between these groups (Figure S4D), suggesting that I-BET did not augment GSIS in vivo at this late stage. Interestingly, the apoptosis of pancreatic β-cells induced by STZ was significantly decreased by I-BET (Figure 6C,D), consistent with the effects seen in INS-1 cells. However, no difference in the β-cell area and islet size was observed (Figure S4E,F). Together, these data indicated that I-BET, though unable to rescue insulin secretion in vivo, was sufficient to prevent inflammation-induced apoptosis and preserve the β-cell area in the T1D model, as seen in vitro upon chronic cytokine exposure.

## 4. Discussion

**Figure 7:**
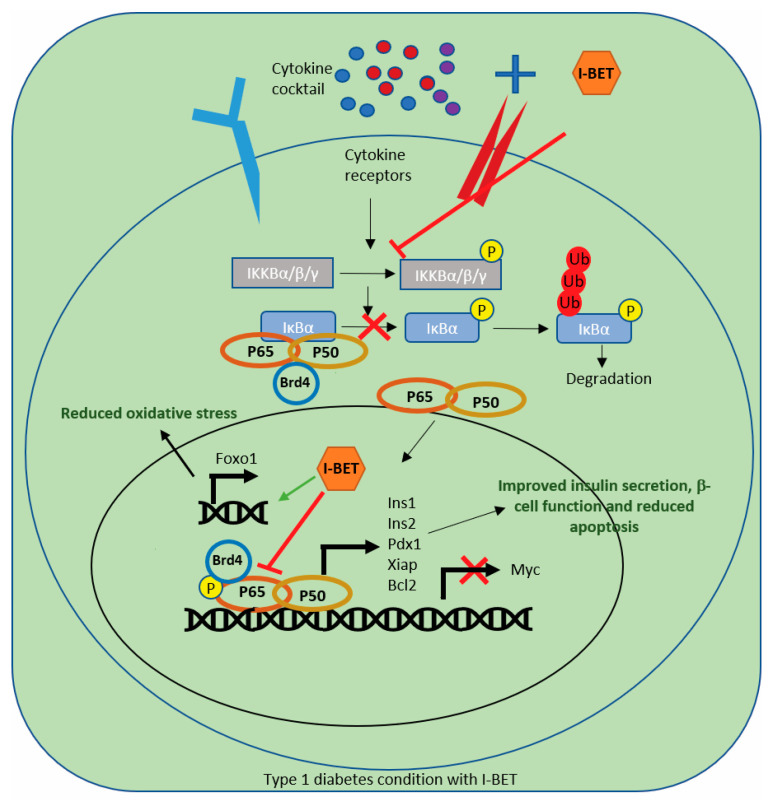
I-BET protects against cytokine-induced apoptosis by antagonizing NF-kB signaling. Under inflammation, cytokines bind to their receptors, leading to the phosphorylation of IKKA/B, which in turn leads to the phosphorylation and degradation of IκBα, relieving p65 inhibition. Phospho-p65 then translocates to the nucleus, interacting with BRD proteins and regulating NF-κB target gene transcription, including the repression of β-cell function-related genes such as INS-1, Ins2, and *Pdx1*, and anti-apoptotic genes (*Xiap),* and the activation of inflammatory genes such as *Myc*, leading to reduced insulin secretion, β-cell dysfunction, and ultimately apoptosis. However, when treated with I-BET, the phosphorylation of IKKA/B, IκBα, and p65 is reduced, inhibiting the binding of BRD to p65, indicating an overall decrease in NF-κB pathway activity, reversing the gene expression alterations, improving β-cell function, and reducing apoptosis. Additionally, I-BET-regulated Foxo1 expression and its downstream target Sod1 reduce oxidative stress.

Inflammation and cytokine-induced β-cell dysfunction and apoptosis are central mechanisms in the pathogenesis of T1D [1,2,3]. However, no targeted therapies are available in clinical practice that protect β-cells from cytokine-induced damage. In this study, we demonstrate that I-BET, a BET inhibitor, specifically affects β-cells by antagonizing the NF-κB pathway, protecting against cytokine-induced dysfunction and cell death both in vitro and in vivo (Figure 7).

Cytokine exposure induces the repression of genes critical for insulin secretion, including *Pdx1* and *MafA,* and severe β-cell dysfunction [53]. Here, we show that BET inhibition improved β-cell function by augmenting *Pdx1*, *MafA*, and *Pax6* gene expression, which is critical for maintaining β-cell secretory function. Interestingly, some genes, such as *Nkx2.2* and *Nkx6.1*, were not responsive to I-BET. This indicates that bromodomain-dependent regulatory pathways in β-cells are specific or have potential differential susceptibility to these genes in I-BET regulation.

NF-κB signaling is the major pathway responsible for β-cell apoptosis [41,42], particularly in T1D pathogenesis. Many studies in various tissues undergoing inflammation demonstrated NF-κB activation that can be modulated by bromodomain proteins and display responsiveness to I-BET suppression [54,55,56,57]. To date, these effects of I-BET have not been tested in β-cells. Brd4, an I-BET target, is known to co-activate NF-κB signaling by interacting with acetylated RelA (p65) to promote its stability and transactivation activity in cancer cells [55,58]. I-BET has also been shown to disrupt this binding in β-cells [43], where it regulates *NOS2* expression. Transcriptomic analysis of islets from NOD mice treated with I-BET revealed differential expression of NF-κB targets [22], suggesting that BET proteins regulate at least a subset of NF-κB target genes in β-cells. Herein, we identify a potential mechanism of I-BET action in protecting β-cells from cytokine-induced dysfunction and apoptosis through inhibiting IKKA/B and IκB-mediated p65 (RelA) phosphorylation which is required for NF-κB translocation and transactivation. Our findings in β-cells are consistent with the effects of BET inhibition in cell types such as B cells, synoviocytes involved in rheumatoid arthritis, and microglia cells [59,60,61]. In addition to regulating phospho-p65 (RelA), there are other potential mechanisms by which I-BET treatment may suppress NF-κB signaling. BET inhibitors can inhibit the binding of Brd4 to acetylated RelA in a dose-dependent manner, a critical step in the formation of the RelA: P-TEFb transcription–activation complex [58,62]. Another mechanism could prevent the formation of RelA-dependent super-enhancers required to activate the cytokine-inflammatory pathways [63]. The identification and regulation of these additional mechanisms warrant future studies.

In addition to NF-κB, I-BET is also shown to regulate the Foxo1 pathway by increasing its expression and its downstream mediator Sod1 (superoxide dismutase 1), which is an anti-oxidant enzyme and protects cells from oxidative stress [64,65]. Foxo1 is crucial for β-cell growth and function in response to various kinds of stress [49]. Tan et al. also show that the knockdown of Brd4 increases *Foxo1* mRNA and protein similar to JQ1 in prostate cancer [66], indicating Brd4-mediated inhibition of Foxo1 transcription. Thus, an increase in Foxo1 function by I-BET reveals its diverse mechanistic insight in preventing apoptosis. I-BET is known to reduce oxidative stress [48] and is likely to play a similar role in mitigating oxidative stress by acting via the Foxo1 signaling pathway as an additional mechanism in preventing cytokine-induced apoptosis of β-cells.

Despite β-cell dysfunction induced by 8 h of cytokine exposure, an increase in apoptosis required prolonged exposure for 24 h, indicating that apoptosis is a late event of cytokine-induced β-cell dysfunction. However, with the onset of cytotoxicity and apoptosis after 24 h cytokine exposure, I-BET could not rescue cytokine-induced β-cell dysfunction, including insulin secretion and related β-cell gene expression. This inability of I-BET to improve β-cell function after prolonged cytokine induction could underlie the modest glycemic response and the failure to rescue the loss of insulin secretion during the GTT in our STZ-diabetic mice. Notably, similar to the in vitro studies, I-BET successfully prevented apoptosis in the STZ-diabetic mice, which may have translational value in early human T1D management. A limitation of the current study is that systemic administration of I-BET to STZ-induced diabetic mice may regulate infiltrating immune cells in the islets and other cell types in ameliorating hyperglycemia. Hence, the in vivo effects of I-BET may not be attributable only to the cell-autonomous effects of I-BET in β-cells. Though the studies in INS-1 cells strongly support the cell-autonomous effects of I-BET in β-cells in vivo, similar experiments in mice with selective genetic deletion of specific BET proteins or the design of specific inhibitors to the individual BET proteins may provide more definitive support.

Global transcriptomic analysis revealed that I-BET prevented many but not all transcriptomic changes induced by cytokine exposure. This could result from the differential dependence of cytokine-induced transcriptional changes on BET-dependent or BET-independent mechanisms or may depend on the specific roles of bromodomain proteins, such as Brd2 or Brd4. Thus, a combination of BET-inhibiting agents may be needed to fully repress the deleterious effects of cytokines. The current study was not designed to determine the relative role of the various BET proteins in the protective effect of I-BETs. However, the rational design of specific BET inhibitors that affect BET proteins differently could prove more efficacious in mitigating β-cell dysfunction and death in immune-mediated diabetes.

## Data Availability

Parts of this study were presented in abstract form at the 80th Scientific Sessions of the American Diabetes Association (https://doi.org/10.2337/db20-2036-P, accessed date 10 June 2024). A non-peer-reviewed previous version of this article was posted on the BioRxiv preprint server (MS ID#: BIORXIV/2020/363408) on 5 November 2020.

## Supplementary Materials

The following supporting information can be downloaded at: https://www.mdpi.com/article/10.3390/cells13131108/s1, Figure S1. Gene expression changes with I-BET at 8 h of cytokine exposure. INS-1 cells were induced with cytokine cocktail for 8 h, and (A–C) Gene expression of (A) *Pax4*, (B) *Nkx2.2*, (C) *Nkx6.1* were measured using qRT-PCR and shown here after normalization to housekeeping gene as a fold change over VC (vehicle control). (D,E) Annexin V-PI staining was analyzed by flow cytometry and represented by a dot plot in D, and its quantitation is shown in E. The data is represented as mean ± sem (n = 5–6), with at least three independent experiments. Statistical significance was calculated using one-way ANOVA; *** *p* < 0.001, ** *p* < 0.01, * *p* < 0.05. Figure 2. I-BET does not rescue cytokine-induced decrease in β-cell function at 24 h. (A–D) Gene expression by RT-qPCR of (A) *Ins1*, (B) *Ins2*, (C) *MafA*, and (D) *Pdx1* are shown after normalization to housekeeping gene as a fold change over VC. (E–G) The secreted insulin from INS-1 cells in basal 2.8 mM glucose (E) and after incubation in indicated glucose concentrations (F) is shown. Insulin secretion is represented as insulin stimulation index (F), a fold change over the respective levels of basal 2.8 mM glucose. (G) Insulin content measured in INS-1 cell lysates normalized to cellular DNA. The data is represented as mean ± sem (n = 3), with at least three independent experiments. Statistical significance was calculated using one-way ANOVA; *** *p* < 0.001, ** *p* < 0.01, * *p* < 0.05. Figure S3. Pathways altered by I-BET in the absence and presence of cytokines. (A) Heatmap representing the differentially expressed genes among all fours groups- VC, IB, CCVC, and CCIB. Red and blue indicate up- and down- regulated normalized Z-score expression. (B) Pathways altered by cytokines (CCVC vs. VC) and by I-BET in the presence of cytokines (CCIB vs CCVC) were determined using David pathway enrichment. Furthermore, they were compared to determine pathways unique and common to both conditions. Figure S4. Effect of I-BET on multiple low dose STZ mouse model of T1D. (A) Body weight (B) Plasma insulin level in fasted mice in indicated groups. (C,D) Glucose tolerance test (GTT) in fasted mice with glucose (C) and insulin (D) levels at indicated time points. (E,F) The beta cell area and islet size were determined in these mice using ImageJ. The data is represented as mean ± sem (n = 9). Statistical significance was calculated using two-way ANOVA; *** *p* < 0.001, ** *p* < 0.01, * *p* < 0.05 for STZ + VC and STZ + I-BET group and # < 0.05 for VC and STZ + VC group. Supplementary Table S1. List of Primers used.
