# Supplementary material for: Bromodomain Protein Inhibition Protects β-Cells from Cytokine-Induced Death and Dysfunction via Antagonism of NF-κB Pathway
Source: Cells. 2024 Jun 26;13(13):1108. doi: 10.3390/cells13131108 (PMC11240345; doi:10.3390/cells13131108)
Supplement: Supplementary file 1 [file cells-13-01108-s001.zip › cells-2957514-supplementary.docx]

Bromodomain Protein Inhibition Protects β-Cells from
Cytokine-Induced Death and Dysfunction via Antagonism of NF-κB Pathway

Vinny Negi ^1^, Jeongkyung Lee ^1^, Varun Mandi ^1^, Joseph Danvers ^1^, Ruya Liu ^1,†^, Eliana M. Perez-Garcia ^1^, Feng Li ^1^, Rajaganapati Jagannathan ^2^, Ping Yang ^1^, Domenic Filingeri ^1^, Amit Kumar ^1^, Ke Ma ^3^, Mousumi Moulik ^2^
and Vijay K. Yechoor ^1,^*

^1^ Diabetes and Beta Cell Biology Center, Division of Endocrinology and Metabolism, University of
Pittsburgh, Pittsburgh, PA 15203, USA; negiv@pitt.edu (V.N.); jkl43@pitt.edu (J.L.);
mandi.varun@medstudent.pitt.edu (V.M.); jed159@pitt.edu (J.D.); ruya.liu@som.umaryland.edu (R.L.);
emperezgarcia@southalabama.edu (E.M.P.-G.); fel43@pitt.edu (F.L.); ivyyang0826@gmail.com (P.Y.);
domfil@pitt.edu (D.F.); amk550@pitt.edu (A.K.)

^2^ Division of Cardiology, Department of Pediatrics, Children’s Hospital of Pittsburgh, University of
Pittsburgh, Pittsburgh, PA 15260, USA; jagan@pitt.edu (R.J.); moulikm@pitt.edu (M.M.)

^3^ Department of Diabetes Complications and Metabolism, Diabetes and Metabolism Research Institute, City of Hope National Medical Center, Duarte, CA 91010, USA; kema@coh.org

***** Correspondence: yechoorv@pitt.edu; +1-412-383-4251

^†^ Current address: Department of Medicine, University of Maryland School of Medicine,
Baltimore, MD 21201, USA.

Online Supplement

**Supplementary Figure S1. Gene expression changes with I-BET at 8h of cytokine exposure.** INS-1 cells were induced with cytokine cocktail for 8 h, and (A–C) Gene expression of (A) *Pax4*, (B) *Nkx2.2*, (C) *Nkx6.1* were measured using qRT-PCR and shown here after normalization to housekeeping gene as a fold change over VC (vehicle control). (D,E) Annexin V-PI staining was analyzed by flow cytometry and represented by a dot plot in D, and its quantitation is shown in E. The data is represented as mean ± sem (n = 5–6), with at least three independent experiments. Statistical significance was calculated using one-way ANOVA; *** *p* < 0.001, ** *p* < 0.01, * *p* < 0.05.


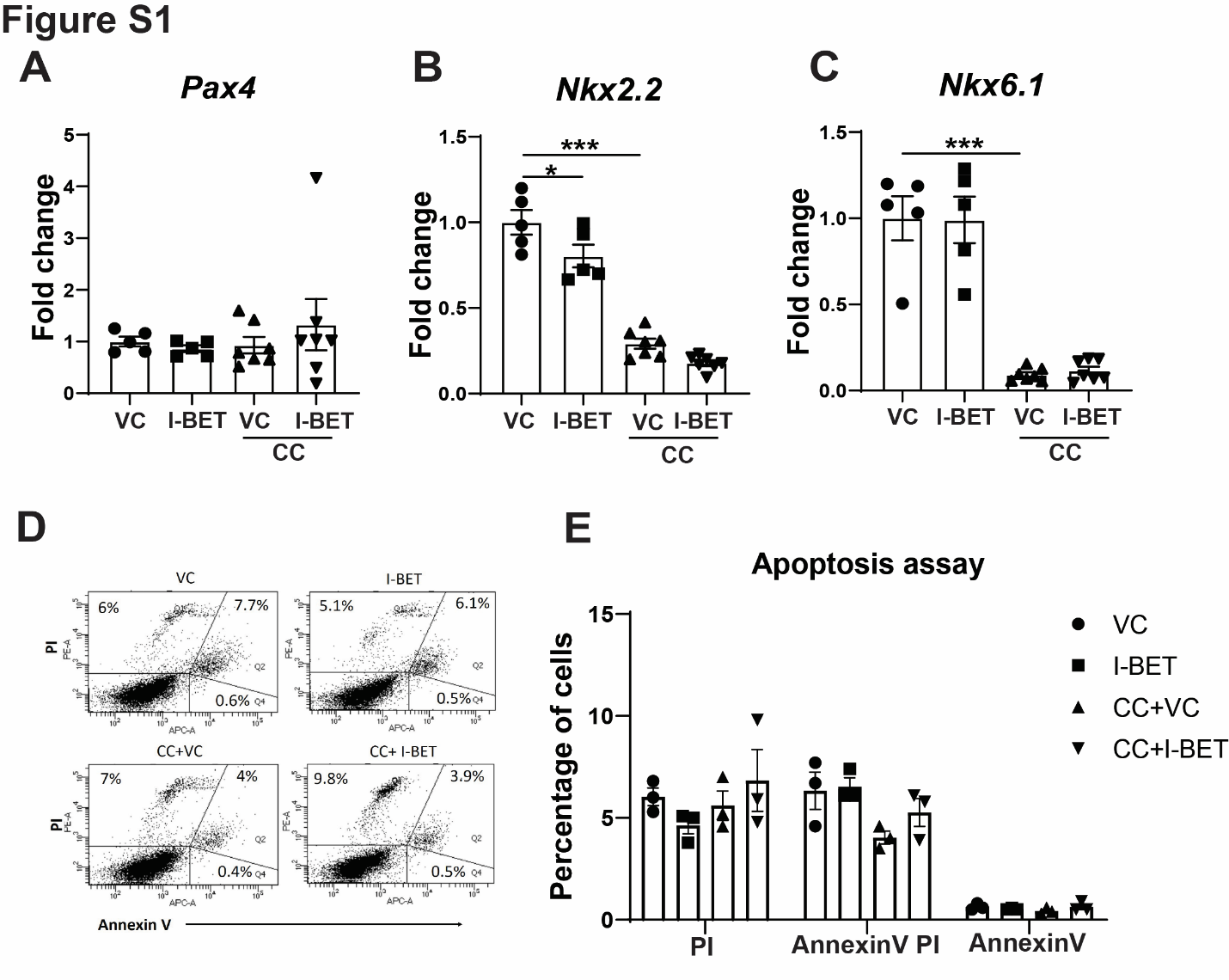


**Supplementary** **Figure S2. I-BET does not rescue cytokine-induced decrease in β-cell function at 24h. (A–D)** Gene expression by RT-qPCR of (**A**) *Ins1*, (**B**) *Ins2*, (**C**) *MafA*, and (D) *Pdx1* are shown after normalization to housekeeping gene as a fold change over VC. (**E–G**) The secreted insulin from INS-1 cells in basal 2.8 mM glucose (E) and after incubation in indicated glucose concentrations (F) is shown. Insulin secretion is represented as insulin stimulation index (F), a fold change over the respective levels of basal 2.8 mM glucose. (**G**) Insulin content measured in INS-1 cell lysates normalized to cellular DNA. The data is represented as mean ± sem (n = 3), with at least three independent experiments. Statistical significance was calculated using one-way ANOVA; *** *p* < 0.001, ** *p* < 0.01, * *p* < 0.05.


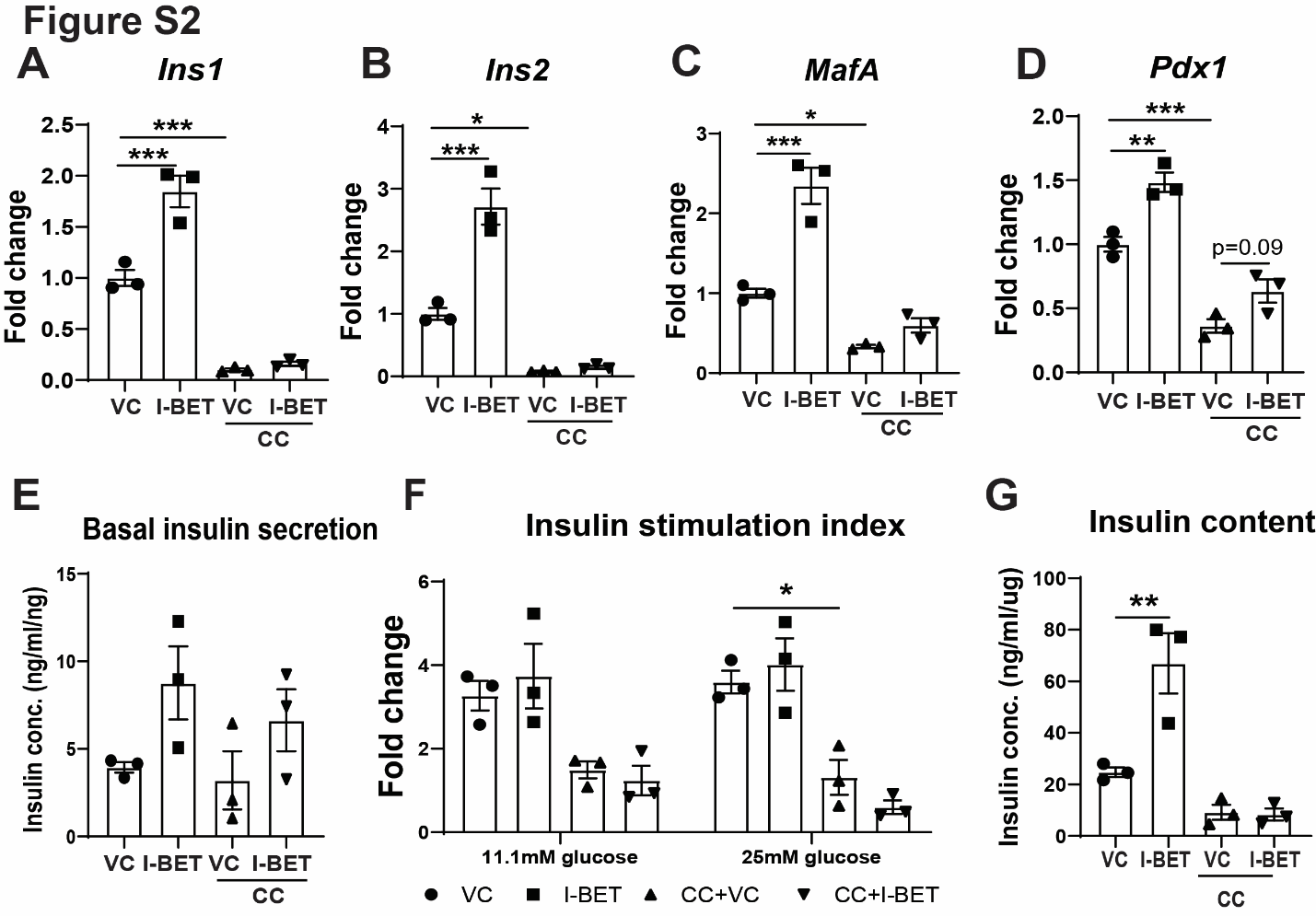


**Supplementary** **Figure S3. Pathways altered by I-BET in the absence and presence of cytokines.** (**A**) Heatmap representing the differentially expressed genes among all fours groups- VC, IB, CCVC, and CCIB. Red and blue indicate up- and down- regulated normalized Z-score expression. (**B**) Pathways altered by cytokines (CCVC vs VC) and by I-BET in the presence of cytokines (CCIB vs CCVC) were determined using David pathway enrichment. Furthermore, they were compared to determine pathways unique and common to both conditions.


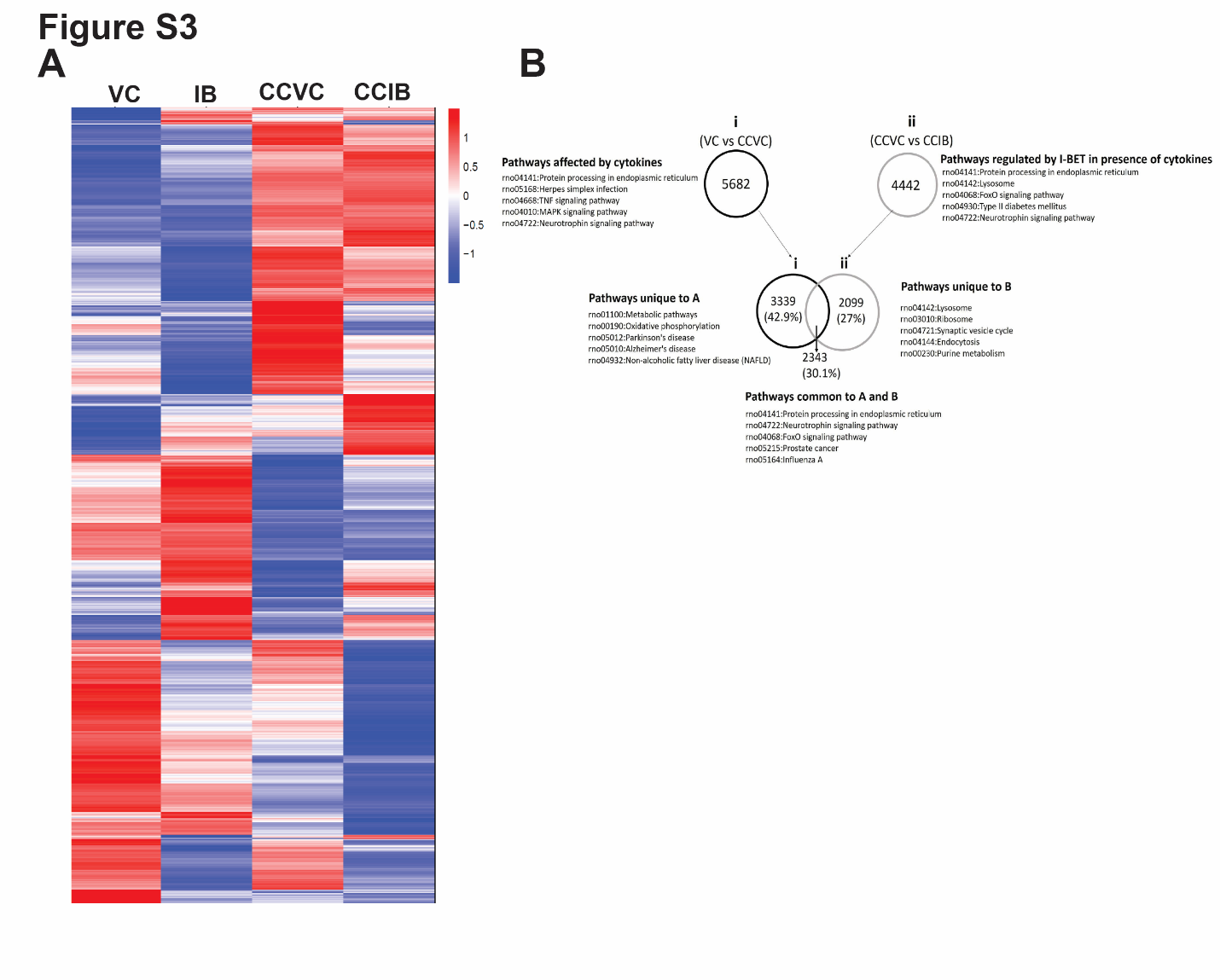


**Supplementary Figure S4.** **Effect of I-BET on multiple low dose STZ mouse model of T1D.** (**A)** Body weight **(B)** Plasma insulin level in fasted mice in indicated groups. **(C-D)** Glucose tolerance test (GTT) in fasted mice with glucose (C) and insulin (D) levels at indicated time points. (**E-F**) The beta cell area and islet size were determined in these mice using ImageJ. The data is represented as mean ± sem (n = 9). Statistical significance was calculated using two-way ANOVA; *** *p* < 0.001, ** *p* < 0.01, * *p* < 0.05 for STZ + VC and STZ + I-BET group and # < 0.05 for VC and STZ + VC group.


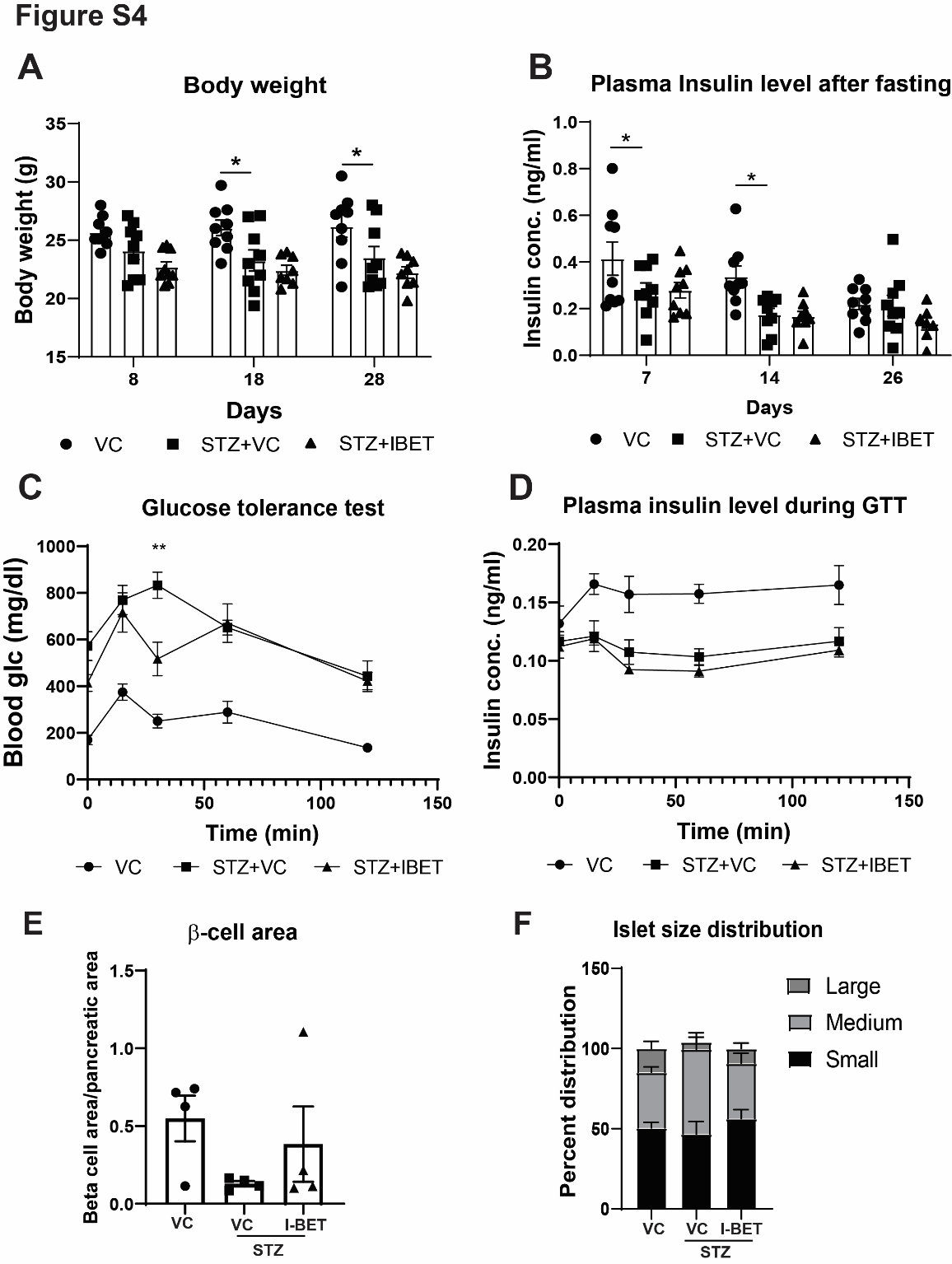


**Supplementary Table S1.** List of Primers used.

| **S.No.** | **Gene name** | **Primer name** | **Sequence** |
| --- | --- | --- | --- |
| 1. | Insulin 1 | rt-Ins1-FP | ACAGCACCTTTGTGGTCCTC |
| 2. |  | rt-Ins1-RP | GTGCAGCACTGATCCACAAT |
| 3. | Insulin 1 | rt-Ins2-FP | TGTGGTTCTCACTTGGTGGA |
| 4. |  | rt-Ins2-RP | ATGCTGGTGCAGCACTGA |
| 5. | Pancreatic and Duodenal Homeobox 1 | rt-Pdx1-FP | AGCTCACGCGTGGAAAAG |
| 6. |  | rt-Pdx1-RP | GTACGGGTCCTCTTATTCTCCTC |
| 7. |  | hu-Pdx1-FP | GGAAAACCCGCTCTCTCAGG |
| 8. |  | hu-Pdx-1-RP | CCAAGGTGGAGTGCTGTAGG |
| 9. | MAF BZIP Transcription Factor A | rt-Mafa-FP | AGCAAGGAGGAGGTCATC |
| 10. |  | rt-Mafa-RP | CGTATTTCTCCTTGTACAGG |
| 11. |  | hu-Mafa-FP | AGAGCGAGAAGTGCCAACTC |
| 12. |  | hu-Mafa-RP | TTGTACAGGTCCCGCTCTTT |
| 13. | Paired Box 6 | rt-Pax6-FP | CCAGTTTTCAGAGCCACGTAT |
| 14. |  | rt-Pax6-RP | ACTCCGCTGTGACTGTTCTG |
| 15. | NK6 Homeobox 1 | rt-Nkx6.1-FP | ATGGGAAGAGAAAACACACCAGAC |
| 16. |  | rt-Nkx6.1-RP | TAATCGTCGTCGTCCTCCTCGTTC |
| 17. | NK2 Homeobox 2 | rt-Nkx2.2-FP | CAGCGACAACCCCTACACTC |
| 18. |  | rt-Nkx2.2-RP | GCTTTGGAGAAGAGCACTCG |
| 19. | Paired Box 4 | rt-Pax4-FP | AGATGTTCCAGTGACACCACA |
| 20. |  | rt-Pax4-RP | CACAGGAAGGAGGGAGTGG |
| 21. | Myc | Rt-Myc-FP | GCTCCTCGCGTTATTTGAAG |
| 22. |  | Rt-Myc-RP | GCATCGTCGTGACTGTCG |
| 23. | Xiap | Rt-Xiap-FP | GCTTGCAAGAGCTGGATTTT |
| 24. |  | Rt-Xiap-RP | TGGCTTCCAATCCGTGAG |
