# Supplementary figures and images for: Bromodomain Protein Inhibition Protects β-Cells from Cytokine-Induced Death and Dysfunction via Antagonism of NF-κB Pathway
Source: Cells. 2024 Jun 26;13(13):1108. doi: 10.3390/cells13131108 (PMC11240345; doi:10.3390/cells13131108)

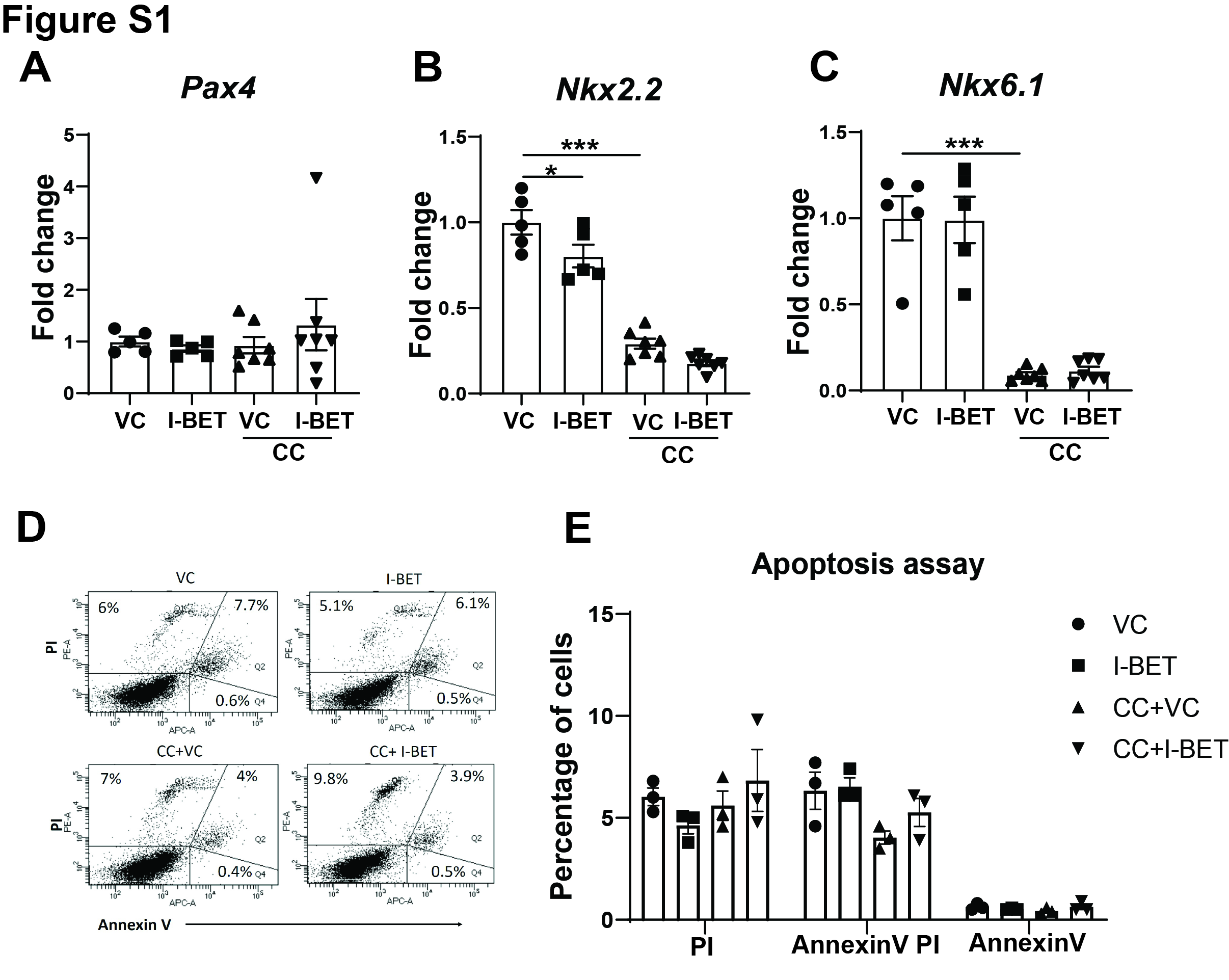

Supplement: Supplementary file 1 [file cells-13-01108-s001.zip › Figure S1.tif]

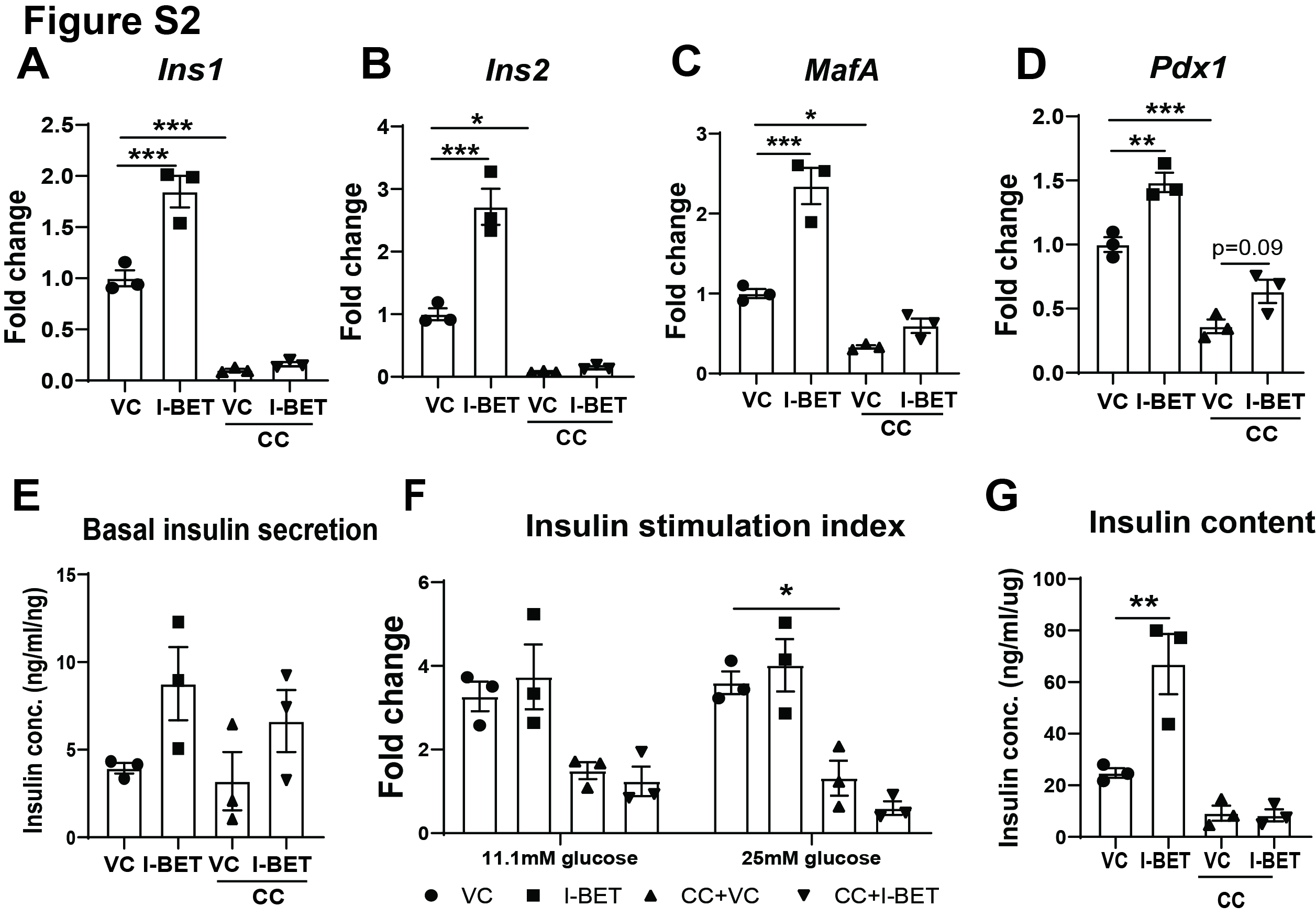

Supplement: Supplementary file 1 [file cells-13-01108-s001.zip › Figure S2.tif]

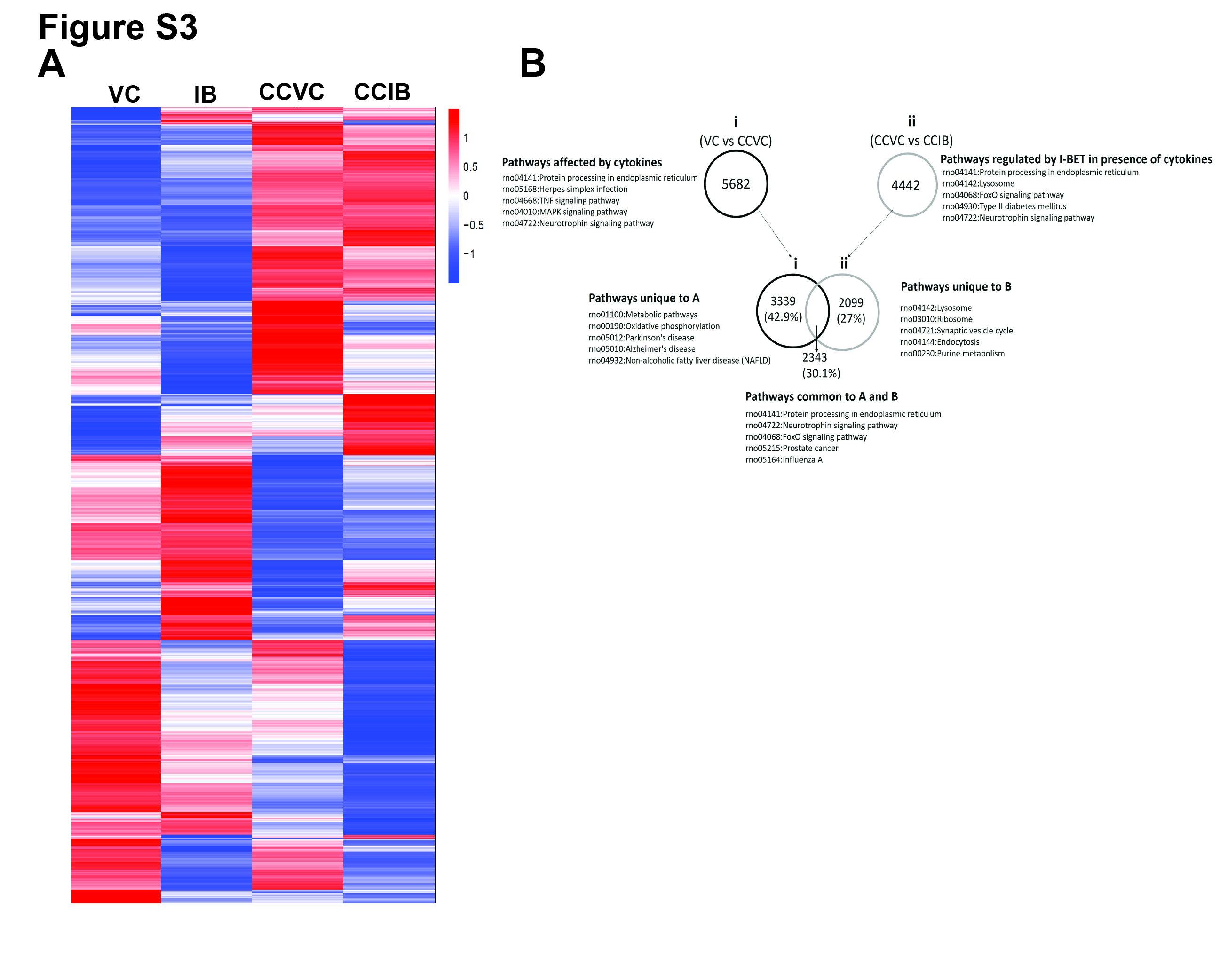

Supplement: Supplementary file 1 [file cells-13-01108-s001.zip › Figure S3.tif]

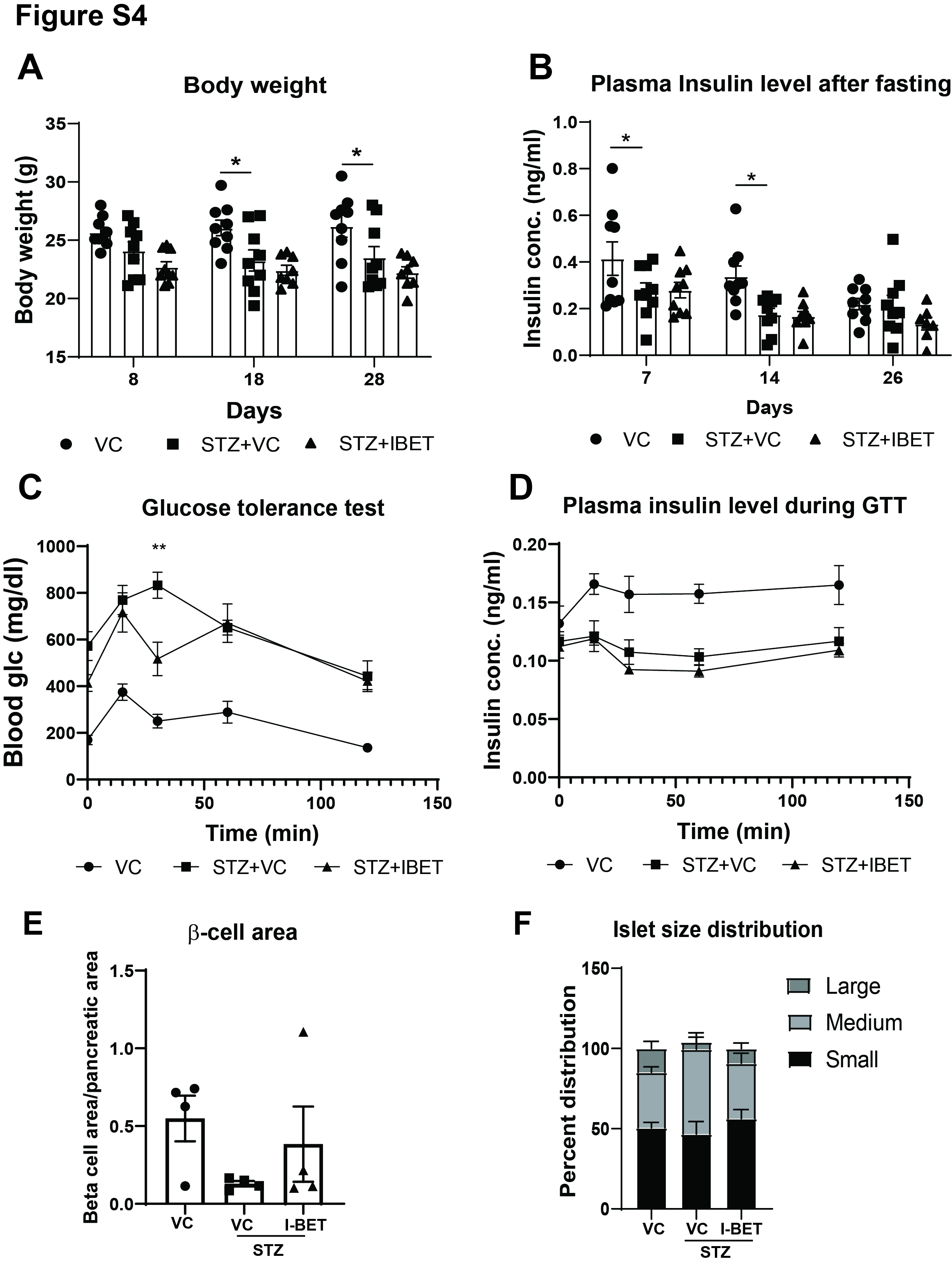

Supplement: Supplementary file 1 [file cells-13-01108-s001.zip › Figure S4.tif]
